# Influenza B Virus Infection Is Enhanced Upon Heterotypic Co-infection With Influenza A Virus

**DOI:** 10.3389/fmicb.2021.631346

**Published:** 2021-02-25

**Authors:** Nicolas Malausse, Sylvie van der Werf, Nadia Naffakh, Sandie Munier

**Affiliations:** ^1^Unité de Génétique Moléculaire des Virus à ARN, Institut Pasteur, CNRS UMR 3569, Université de Paris, Paris, France; ^2^Université de Paris, Sorbonne Paris Cité, Paris, France

**Keywords:** influenza virus, co-infection, heterotypic, influenza B virus, viral interference

## Abstract

Homotypic co-infections with influenza viruses are described to increase genetic population diversity, to drive viral evolution and to allow genetic complementation. Less is known about heterotypic co-infections between influenza A (IAV) and influenza B (IBV) viruses. Previous publications showed that IAV replication was suppressed upon co-infection with IBV. However, the effect of heterotypic co-infections on IBV replication was not investigated. To do so, we produced by reverse genetics a pair of replication-competent recombinant IAV (A/WSN/33) and IBV (B/Brisbane/60/2008) expressing a GFP and mCherry fluorescent reporter, respectively. A549 cells were infected simultaneously or 1 h apart at a high MOI with IAV-GFP or IBV-mCherry and the fluorescence was measured at 6 h post-infection by flow cytometry. Unexpectedly, we observed that IBV-mCherry infection was enhanced upon co-infection with IAV-GFP, and more strongly so when IAV was added 1 h prior to IBV. The same effect was observed with wild-type viruses and with various strains of IAV. Using UV-inactivated IAV or type-specific antiviral compounds, we showed that the enhancing effect of IAV infection on IBV infection was dependent on transcription/replication of the IAV genome. Our results, taken with available data in the literature, support the hypothesis that the presence of IAV proteins can enhance IBV genome expression and/or complement IBV defective particles.

## Introduction

Influenza A (IAV) and influenza B viruses (IBV) are responsible for seasonal epidemics that cause significant mortality and morbidity in humans each year. Two IAV subtypes, A(H1N1)pdm09 and A(H3N2), and two IBV lineages, B/Yamagata and B/Victoria, are currently co-circulating in the human population (Krammer et al., 2018). The circulation pattern differs depending on the influenza season; indeed, the predominant circulating virus may belong to one or the other IAV subtype or IBV lineage. Influenza B infections generally account for approximately 25% of cases annually and usually peak later than IAV infections during seasonal epidemics (Caini et al., 2019). The clinical severity of IBV infections is equivalent to that of IAV (Su et al., 2014), with a higher prevalence of IBV in the pediatric population.

IAV and IBV are described to co-circulate during seasonal epidemics among other respiratory viruses (Nickbakhsh et al., 2019). Infection by more than one type of virus is described as a co-infection, with the specific term superinfection being used when one virus infects the host or the cell before a second superinfecting virus. Co-infections can lead to virus-virus interactions which can in turn alter viral replication, disease severity and disease epidemiology (reviewed in Kumar et al., 2018; Saade et al., 2020). Despite several studies that have investigated the frequency of homotypic or heterotypic influenza co-infections in human respiratory samples, co-infections with IAV and IBV have only occasionally been reported *in vivo* (reviewed in Pérez-García et al., 2016; Gregianini et al., 2019). A retrospective case-control study from flu seasons 2009 to 2018 in Brazil found that the frequency of heterotypic co-infections was less than 0.4% annually in the years during which co-infections were detected (Gregianini et al., 2019). However, in 2017, the frequency of heterotypic co-infections reached 1.3% and was associated with a high frequency of A(H3N2) and IBV co-circulation, thus increasing the chance of co-infection (Gregianini et al., 2019). In this context, the clinical significance of mixed influenza virus infection is not well understood, and its relation to the disease severity is unclear. The outcome of co-infections was found to be associated with increased severity in some studies (Gregianini et al., 2019) but not in others (Pérez-García et al., 2016).

IAV and IBV both belong to the *Orthomyxoviridae* family and share close phylogenic relationship. Their genomes consist of eight single-stranded, negative-sense viral RNA (vRNA) segments, each bound to oligomers of nucleoprotein (NP) and associated with the three subunits of the viral polymerase complex (PB2, PB1, and PA) to form viral ribonucleoproteins (vRNPs) (Krammer et al., 2018). During the viral life cycle, vRNPs enter the host cell nucleus where they are transcribed into capped and polyadenylated viral mRNAs by a cap-snatching mechanism. Upon translation and nuclear import of vRNP components, new rounds of transcription/replication take place. Replication of vRNPs occurs via the synthesis of full-length complementary RNAs which then serve as templates for the synthesis of vRNAs. Newly synthetized vRNPs are then exported in the cytoplasm and transported to the sites of viral assembly where the correct set of eight segments is incorporated into progeny virions (Dou et al., 2018).

Homotypic co-infections with influenza viruses are essential to increase genetic diversification, to drive viral evolution and to allow genetic complementation. Less is known about heterotypic co-infections between IAV and IBV. Because of the segmented nature of their genomes, co-infection of the same cell by several viral particles can lead to genetic reassortment, i.e., the exchange of gene segments between co-infecting viruses (Lowen, 2017). Although intratypic reassortments frequently occur between IAV subtypes or IBV lineages *in vivo*, intertypic reassortments between IAV and IBV have never been detected in nature or successfully generated *in vitro* (McCullers et al., 2004; Baker et al., 2014; Krammer et al., 2018), most likely because of incompatible protein functions and of incompatible packaging signals between the IAV and IBV vRNPs (Muster et al., 1991; Baker et al., 2014).

Several reports have indicated that, upon co-infection of cells *in vitro*, IBV impairs the replication of IAV, a phenomenon called intertypic or heterotypic interference (Tobita and Ohori, 1979; Mikheeva and Ghendon, 1982; Kaverin et al., 1983; Aoki et al., 1984). Mechanistically, it has been shown that the NP of IBV (NP_B_) can inhibit IAV polymerase activity through binding to its type A counterpart (NP_A_) thus disrupting interaction between NP_A_ and PB2, preventing IAV polymerase complex formation and ultimately leading to the growth suppression of co-infecting IAV (Wanitchang et al., 2012; Jaru-ampornpan et al., 2014; Narkpuk et al., 2017). Other mechanisms may also contribute to intertypic interference such as inefficient assembly/functionality of a heterotypic polymerase complex (Iwatsuki-Horimoto et al., 2008; Wunderlich et al., 2010).

Most studies so far have focused on IBV interference on IAV replication, and have suggested that IAV interference on IBV infection was less pronounced (Tobita and Ohori, 1979; Mikheeva and Ghendon, 1982; Kaverin et al., 1983; Aoki et al., 1984). Here, in order to gain further insight into the mutual interference between IAV and IBV, we developed an original system using recombinant viruses harboring a fluorescent reporter in co-infection experiments and analyzed the outcome using flow cytometry on infected cells. Unexpectedly, we observed a significantly enhanced infection of IBV upon co-infection with IAV, whether IAV infection was initiated before, simultaneously or after IBV infection. This effect was confirmed with wild-type viruses and various strains of IAV. We further showed that UV-inactivation or inhibition of IAV transcription/replication with specific drugs abolished the capacity of IAV co-infection to enhance IBV infection.

## Materials and Methods

### Cells, Drugs and Viruses

Human embryonic kidney 293T (ATCC^®^ CRL-3216) and human alveolar epithelial A549 (ATCC^®^ CCL-185) cells were grown in Dulbecco’s modified Eagle’s medium (DMEM) supplemented with 10% fetal calf serum (FCS). Madin-Darby Canine Kidney MDCK (ATCC^®^ CCL-34) cells were grown in Modified Eagle’s Medium (MEM) supplemented with 5% FCS. Nucleozin (Sigma-Aldrich) and pimodivir (CliniSciences) were added to the medium (1 μM and 50 nM, respectively) at the time of infection. The A/WSN/33 (H1N1), A/PR/8/34 (H1N1), A549-adapted A/Bretagne/7608/2009 (H1N1pdm09) and B/Brisbane/60/2008 (B/Victoria) viruses were produced by reverse genetics as described in Fodor et al. (1999), Biquand et al. (2017), and Nogales et al. (2017) and amplified at a multiplicity of infection (MOI) of 0.0001 on MDCK cells. A/WSN/33 expressing GFP (referred to as IAV-GFP) and B/Brisbane/60/2008 expressing mCherry (referred to as IBV-mCherry) were produced by reverse genetics using a modified PB2 reverse genetics plasmid as described below. 293T and MDCK cells were used for the production of recombinant viruses by reverse genetics. MDCK cells were used for the amplification and titration of viral stocks. All co-infections experiments were performed on A549 cells as they are more relevant than MDCK cells for influenza infection experiments.

### Plasmids

**FIGURE 1:**
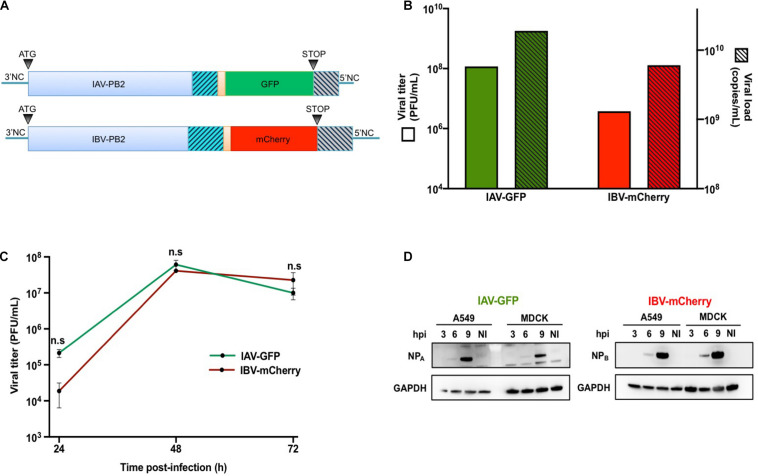
Production and characterization of IAV-GFP and IBV-mCherry recombinant viruses. **(A)** Schematic representation of the IAV-PB2-GFP and IBV-PB2-mCherry segments. PB2: PB2 coding sequence from A/WSN/33 (760 aa) or B/Brisbane/60/2008 (770 aa) viral strains. Orange region: sequence encoding porcine teschovirus-1 2A peptide (22 aa) with AAA linker. GFP/mCherry: flu-codon-optimized sequence encoding the GFP (240 aa) or mCherry (236 aa) fluorescent protein. Gray hatched region: duplication of the last 109 nt (IAV) or 153 nt (IBV) coding for the PB2 protein. Blue hatched region: C-terminal region of PB2 in which silent (non-coding) nucleotide changes were introduced. 3′NC (27 nt for IAV and 23 nt for IBV) and 5′NC (34 nt for IAV and 60 nt for IBV): non-coding regions of the PB2 segment. **(B)** The IAV-GFP and IBV-mCherry infectious titers were determined by plaque assay and expressed in Plaque Forming Units (PFU) per mL (green and red solid bars, respectively; left axis). The corresponding titers in physical particles were determined by RT-qPCR targeting the M (for IAV) and HA (for IBV) genomic segments, and expressed in copy numbers per mL (green and red hatched bars, respectively, right axis). **(C)** MDCK cells were infected with IAV-GFP or IBV-mCherry at a MOI of 0.001 PFU/cell and viral titers in the supernatants collected at 24, 48, and 72 h post-infection were determined by plaque assay. The mean ± S.D. of biological duplicates is shown. Two-way ANOVA test: multiple comparison, Tukey test, α = 0.05, n.s, not significant. **(D)** A549 or MDCK cells were infected with IAV-GFP or IBV-mCherry at a MOI of 3 PFU/cell and the cell lysates prepared at 3, 6, or 9 h post-infection (hpi) were analyzed by Western-blotting using an antibody targeting the nucleoprotein of IAV (NP_A_) or IBV (NP_B_), in parallel with an antibody targeting the GAPDH. NI: not infected.

The monodirectional reverse genetics pPolI-WSN-PB2-2A-GFP plasmid was obtained by subcloning a sequence encoding the 2A peptide from porcine teschovirus-1 followed by a flu-codon-optimized GFP coding sequence into the pPolI-WSN-PB2-2A-Nanoluc plasmid described in Diot et al. (2016) (Figure 1A). The bidirectional reverse genetics plasmid pDP2002-Bris-PB2 was modified using overlapping PCR and synthetic genes in order to duplicate the last 153 nucleotides of the PB2 open reading frame and introduce silent mutations in the region actually encoding the PB2 protein followed by NotI / SpeI restriction sites. The pDP2002-Bris-PB2-2A-mCherry was obtained by subcloning a sequence encoding the 2A peptide from porcine teschovirus-1 followed by a flu-codon-optimized mCherry coding sequence between the NotI / SpeI restriction sites (Figure 1A). All constructs were verified by Sanger sequencing.

### Production of Recombinant Viruses by Reverse Genetics

For IAV, the eight pPolI-WSN-PB2, -PB1, -PA, -HA, -NP, -NA, -NS, -M, and four pcDNA3.1-WSN-PB2, -PB1, -PA, -NP plasmids (0.5 μg of each) were co-transfected into a sub-confluent monolayer of co-cultivated 293T and MDCK cells (4 × 10^5^ and 3 × 10^5^ cells, respectively, seeded in a 6-well plate) using 10 μL of FuGENE^®^ HD transfection reagent (Promega) with 90 μL of Opti-MEM^®^ (Gibco). After 24 h of incubation at 35°C, cells were washed twice with DMEM and incubated in DMEM containing 1 μg/mL of tosyl-phenylalanine chloromethyl ketone (TPCK)-treated trypsin (Sigma-Aldrich) for 48 h at 35°C. Supernatants were harvested and clarified by centrifugation 5 min at 2,500 g before being aliquoted and stored at −80°C. The same protocol was used for IBV using the eight pDP2002-Bris-PB2, -PB1, -PA, -HA, -NP, -NA, -NS, -M bidirectional plasmids and the pCI-Bris-PB2 plasmid (1 μg of each) with 24 μL of FuGENE^®^ HD and 76 μL of Opti-MEM^®^. The efficiency of reverse genetics was evaluated by titrating the supernatant on MDCK cells by plaque assay as described in Matrosovich et al. (2006). Recombinant viruses were agarose plaque-purified and amplified at a MOI of 0.0001 on MDCK cells in DMEM containing 1 μg/mL of TPCK-treated trypsin for 3 days at 35°C.

### Quantification of Viral RNAs

Viral RNAs were extracted using the QIamp Viral RNA kit (Qiagen) from 140 μL of viral stocks. Viral RNAs were quantified by RT-qPCR using the SuperScript III Platinium One-Step qRT-PCR kit (Invitrogen). For IAV, the M segment was detected using the following primers and probe: 5′-CTTCTAACCGAGGTCGAAACGTA-3′, 5′-GGTGA CAGGATTGGTCTTGTCTTTA-3′ and 5′(HEX)-TCAGGCCCC CTCAAAGCCGAG-(BHQ-1)3′. For IBV, the HA segment was detected using the following primers and probes: 5′-AC CCTACARAMTTGGAACYTCAGG-3′, 5′-ACAGCCCAAGCC CAAGCCATTGTTG-3′, 5′(FAM)-AAATCCAATTTTRCTGGT AG-(BHQ-1)3′ and 5′(FAM)-AATCCGATTTTRCTGGTAG-(BHQ-1)3′. Standard curves were obtained by subjecting *in vitro*-transcribed IAV-M or IBV-HA vRNAs to RT-qPCR in parallel. The following program was used on a Light Cycler480 instrument (Roche): 45°C for 15 min, 95°C for 3 min, 50 cycles of 95°C for 10 s, 55°C for 10 s, and 72°C for 20 s, then 40°C for 30 s.

### Viral Replication Kinetics

MDCK cells were seeded on 96-well plates with 2 × 10^4^ cells/well. One day later, cells were infected at a MOI of 0.001 with 50 μL of inoculum. Following 1 h of adsorption at 35°C, cells were washed twice with DMEM and incubated with 100 μL of DMEM containing 0.5 μg/mL of TPCK-treated trypsin. Plates were then incubated at 35°C for 24, 48, or 72 h. Viral supernatants collected at different time points were titrated on MDCK cells by plaque assay as described in Matrosovich et al. (2006).

### Western-Blot Analysis

A549 and MDCK cells were seeded on 96-well plates with 2.5 × 10^4^ cells/well. One day later, cells were infected at a MOI of 3 with 50 μL of inoculum. Following 1 h of adsorption at 35°C, cells were washed twice with DMEM and incubated with 100 μL of DMEM containing 10% FCS. Plates were then incubated at 35°C for 3, 6, or 9 h. Protein extracts were prepared in Laemmli buffer, separated on 4–12% NuPAGE Bis-Tris gel (Invitrogen) and transferred onto polyvinylidene fluoride (PVDF) membranes (Hybond^®^, Amersham). Immunoblot membranes were incubated with primary antibodies directed against NP (AAH5, Abcam, 1/5,000 for IAV; B017, Abcam, 1/1,000 for IBV) or GAPDH (Pierce) and revealed with peroxidase-conjugated secondary antibodies (GE Healthcare) and the ECL2 substrate (Thermo Fisher Scientific). The chemiluminescence signals were acquired using a G-Box and the GeneSnap software (SynGene).

### Virus Inactivation

Four hundred microliters of IAV-GFP in a 35 mm dish were exposed to UV irradiation (8 cycles of 1 mJ/cm^2^; UVC 500 UV Crosslinker, GE Life Sciences). Virus inactivation was assessed by absence of detectable fluorescent cells upon infection of A549 cells and absence of plaque upon titration by plaque assay.

### Detection of Heterotypic Co-Infections by Flow Cytometry

**FIGURE 2:**
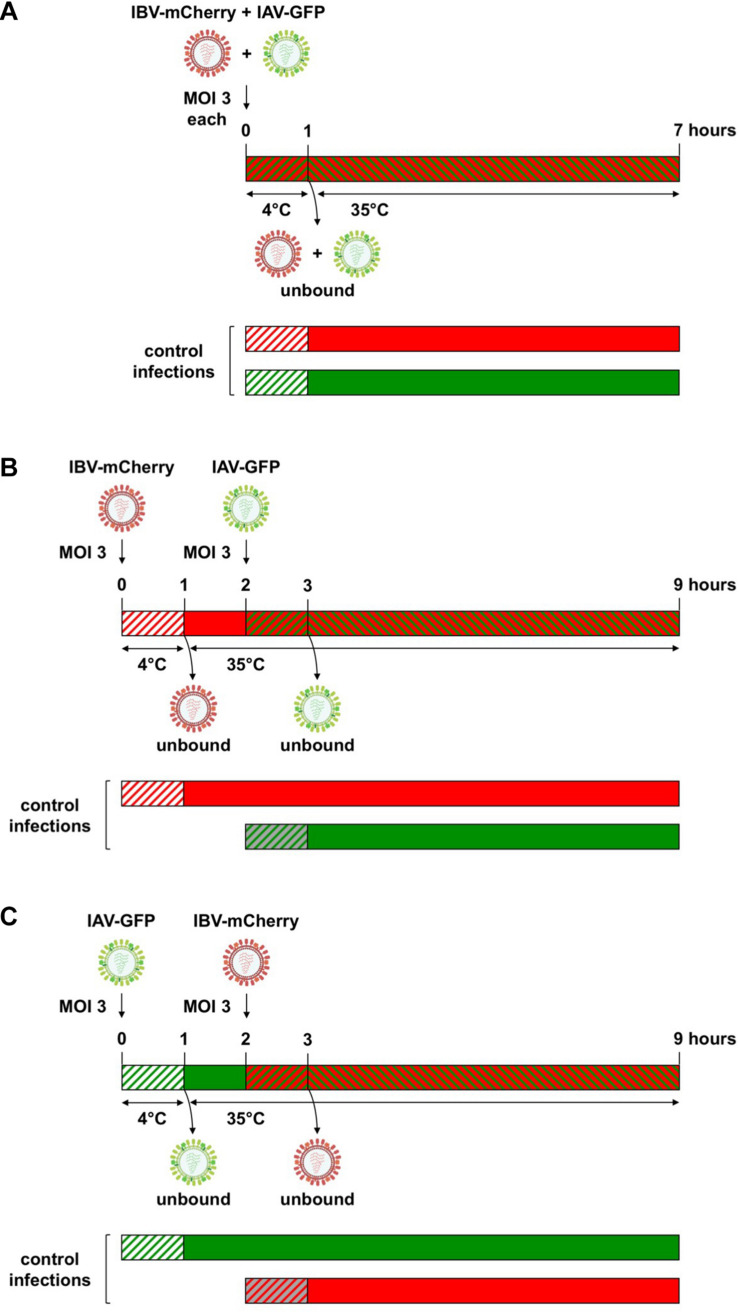
Experimental setup for heterotypic co-infections between IAV and IBV. **(A)** For simultaneous co-infections, A549 cells were infected with a mixture of IAV-GFP and IBV-mCherry using a MOI of 3 for each virus. After 1 h of adsorption at 4°C, unbound viruses were removed by washing and cells were further incubated for 6 h at 35°C. For sequential co-infections, IBV-mCherry **(B)** or IAV-GFP **(C)** were first added on A549 at a MOI of 3 for 1 h at 4°C, unbound viruses were removed by washing and cells were incubated for 1 h at 35°C to allow virus entry. At 1 hpi, cells were infected at a MOI of 3 with IAV-GFP **(B)** or IBV-mCherry **(C)**. After 1 h of adsorption at 35°C, unbound viruses were removed by washing and cells were further incubated for 6 h at 35°C. Cells were then fixed and the fluorescence was measured by flow cytometry. In parallel, control infections were performed where one of the co-infecting viruses was omitted (either the first or the second one). Green and red hatched bars: IAV-GFP + IBV-mCherry; green hatched or solid bars: IAV-GFP; red hatched or solid bars: IBV-mCherry; hatched bars on white background: incubation at 4°C; hatched bars on gray or colored background and solid bars: incubation at 35°C. For IBV-mCherry, the indicated MOI refers to the stock presented in Figure 1 (MOI of 3 PFU/cell, corresponding to ∼ 5 × 10^3^ physical particles/cell). For other stocks of IBV-mCherry, an amount of ∼5 × 10^3^ physical particles/cell was used to obtain between 20 and 60% of mCherry-positive cells at 6 h post-infection.

A549 cells were seeded on 96-well plates with 2 × 10^4^ cells/well. One day later, cells were infected at a high MOI with 50 μL of (i) a mixture of IAV-GFP and IBV-mCherry (Figure 2A), (ii) IBV-mCherry (Figure 2B), or (iii) IAV-GFP (Figure 2C). Following 1 h of adsorption at 4°C, cells were washed twice with cold DMEM and incubated with 100 μL of Opti-MEM^®^ for 1 h at 35°C. Cells were then washed twice with DMEM and 50 μL of (i) DMEM, (ii) IAV-GFP, or (iii) IBV-mCherry at a high MOI were added. Following 1 h of adsorption at 35°C, cells were washed twice with DMEM and incubated with 100 μL of Opti-MEM^®^ for 6 h at 35°C. Infected cells were detached with 30 μL of trypsin (Gibco) for 15 min at 37°C, incubated with 120 μL of DMEM containing 5% FCS and then transferred to 150 μL of PBS containing 8% formaldehyde. After 20 min of incubation at room temperature, fixed cells were centrifuged 5 min at 1,500 g, washed once in PBS and resuspended in 200 μL of PBS. Fluorescence was measured by flow cytometry (Attune NxT, Thermo Fisher Scientific). The MOI was 3 Plaque Forming Units (PFU)/cell for IAV-GFP and between 3 and 25 PFU/cell (corresponding to ∼ 5 × 10^3^ physical particles/cell) for IBV-mCherry, depending on the viral stock, to obtain 50–80% GFP-positive and 20–60% mCherry-positive cells, respectively, at 6 h post-infection. All experiments with fluorescent viruses were carried out in biological triplicates.

The same protocol was applied with IAV and IBV wild-type viruses, except that infected cells were detected using flow cytometry upon NP-labeling. Briefly, A549 cells were seeded on 24-well plates with 2 × 10^5^ cells/well and infected as described above. After the last centrifugation step, cells were permeabilized for 6 min using PBS-1% Triton X100, washed three times with PBS-1% FCS, and incubated 10 min with PBS-1% FCS. Cells were then labeled with primary antibody directed against NP_A_ (AAH5, Abcam, 1/5,000) or NP_B_ (B017, Abcam, 1/1,000) for 1 h at 4°C followed with AF488-secondary antibody (Thermo Fisher Scientific, 1/250) for 1 h at 4°C. After the last centrifugation step, cells were resuspended in PBS and fluorescence was measured by flow cytometry.

### Statistical Analysis

Statistical analysis was performed using the GraphPad Prism 8 software. Data were expressed as the mean ± standard deviation (SD). The Shapiro-Wilk’s test was used to assess the normal distribution of the data before using parametric tests. Statistical significance was determined by two-way analysis of variance (ANOVA) with Dunn-Sidak’s multiple-comparison test, or three-way ANOVA with Tukey’s multiple-comparison test, as specified in the figure legends.

## Results

### Production and Characterization of IAV-GFP and IBV-mCherry Recombinant Viruses

We used flow cytometry to quantitatively evaluate viral interference upon heterotypic co-infections with IAV and IBV. To this end, we produced a pair of recombinant IAV (A/WSN/33) and IBV (B/Brisbane/60/2008) expressing a fluorescent protein from the PB2 segment, using a similar approach as described in Tran et al. (2013), Fulton et al. (2015), and Diot et al. (2016). Briefly, a flu-codon-optimized sequence encoding GFP or mCherry was inserted at the C-terminal end of the PB2 coding sequence from IAV or IBV, respectively, downstream of a 2A proteolytic cleavage site from porcine teschovirus-1. To ensure the conservation of the packaging signals at the 5′ end of the viral genomic segment, the last 109 or 153 nucleotides coding for the PB2 protein of IAV or IBV, respectively, were duplicated and inserted after the stop codon of the fluorescent protein. Silent mutations were introduced in the PB2 coding sequence to avoid an exact sequence duplication and prevent genetic instability of the recombinant viruses (Figure 1A).

IAV-GFP and IBV-mCherry were rescued by reverse genetics and viral stocks were obtained after amplification at a MOI of 0.0001 on MDCK cells from plaque-purified viruses. Both IAV and IBV reporter viruses grew to high viral titers (1.2 × 10^8^ and 3.8 × 10^6^ PFU/mL, respectively, Figure 1B, solid bars), close to the ones obtained with their wild-type counterparts (∼10^7^ PFU/mL, data not shown). The concentration of physical viral particles was estimated by RT-qPCR quantification of the M and HA segment copy numbers for IAV and IBV, respectively (Figure 1B, hatched bars). The physical to infectious particles ratio was about 160:1 and 1,600:1 for IAV-GFP and IBV-mCherry, respectively, indicating a higher proportion of non-infectious particles in the IBV-mCherry viral stock.

The growth characteristics of IAV-GFP and IBV-mCherry were next assessed on MDCK and A549 cells. Under multicycle growth conditions on MDCK cells, both IAV-GFP and IBV-mCherry grew efficiently and at a similar rate, reaching a peak of 6 × 10^7^ PFU/mL and 4 × 10^7^ PFU/mL, respectively, at 48 h post-infection (hpi) (Figure 1C). Under the same conditions, IBV-mCherry grew very poorly on A549 cells (data not shown). However, under single-cycle growth conditions, both IAV-GFP and IBV-mCherry were found to infect A549 cells efficiently. Indeed, when A549 cells were infected at a MOI of 3 and NP expression was assessed in cell lysates prepared at 3, 6, and 9 hpi, both the NP_A_ and NP_B_ proteins were detectable at 6 hpi and were strongly expressed at 9 hpi, a pattern very similar to the one observed in MDCK cells (Figure 1D). A549 infected at a high MOI with IAV-GFP or IBV-mCherry were also analyzed at 6 hpi by flow cytometry, and between 52–86% and 16–57% of the cells expressed GFP or mCherry fluorescent proteins, respectively (see below). Based on these data, we chose to use A549 cells under conditions of single-cycle infection to further explore the viral interference between IAV-GFP and IBV-mCherry.

### Mutual Effects of Influenza A and B Virus Upon Heterotypic Co-infection

**FIGURE 3:**
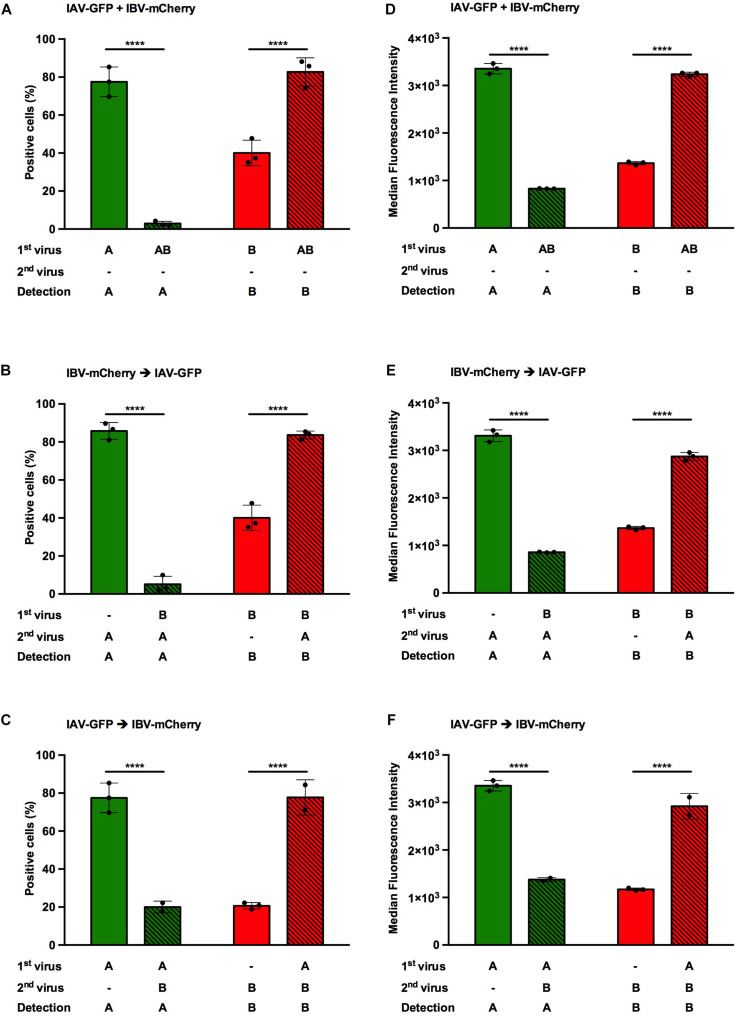
Effect of heterotypic co-infections between IAV and IBV. A549 cells were co-infected, either simultaneously **(A,D)** or sequentially **(B,E,C,F)**, with IAV-GFP and IBV-mCherry at a MOI of 3 PFU/cell for each virus and analyzed 6 h after the addition of the last virus using flow cytometry. **(A–C)** The solid bars represent the proportion of fluorescent cells expressing GFP (in green) or mCherry (in red) upon control infection with IAV-GFP or IBV-mCherry alone. The hatched bars represent the proportion of fluorescent cells expressing GFP (in green) or mCherry (in red) upon co-infections. **(D–F)** The corresponding median fluorescence intensities are shown. **(A,D)** Simultaneous co-infection with IAV-GFP and IBV-mCherry viruses. **(B,E)** Primary infection with IBV-mCherry followed 1 hpi with IAV-GFP infection. **(C,F)** Primary infection with IAV-GFP followed 1 hpi with IBV-mCherry infection. One experiment representative of three independent experiments performed in triplicate is shown (see Supplementary Figure S1). The mean ± S.D. of biological triplicates is shown. Two-way ANOVA test: multiple comparison, Dunn-Sidak test, α = 0.05; ****: adjusted *p* ≤ 0.0001.

To assess the mutual effects of IAV and IBV upon co-infection, we co-infected A549 cells with IAV-GFP and IBV-mCherry by adding the two viruses either simultaneously or 1 h apart, as described in the “Materials and Methods” section and in Figure 2. For the purpose of simplicity, the term co-infection will be used below for both simultaneous and temporally separated co-infections. In parallel, control mock-infections were performed where one or the other co-infecting virus was omitted. Cells were then fixed and the fluorescence was measured by flow cytometry (Figure 3).

When cells were infected with IAV-GFP alone, the proportion of GFP-positive cells varied from 52 to 86%, depending on the experiment (Figure 3 and Supplementary Figure S1, green solid bars). When IBV-mCherry was added, regardless the timing of addition, the proportion of GFP-positive cells was significantly decreased with a proportion of positive cells between 0.2 and 20%, as expected from previous reports (Tobita and Ohori, 1979; Mikheeva and Ghendon, 1982; Kaverin et al., 1983; Aoki et al., 1984; Wanitchang et al., 2012; Figure 3 and Supplementary Figure S1, green hatched bars). The inhibitory effect of IBV on IAV infection was more pronounced if IBV-mCherry was added 1 h before (17-fold decrease, Figure 3B) or at the same time (27-fold decrease, Figure 3A) than 1 h after (3.9-fold decrease, Figure 3C) IAV-GFP. The same trend was observed when the mean fluorescence intensities were recorded (Figures 3D–F). Similar results were obtained in two other experiments (Supplementary Figure S1).

When cells were infected with IBV-mCherry alone, the proportion of mCherry-positive cells varied from 16 to 57%, depending on the experiment (Figure 3 and Supplementary Figure S1, red solid bars). Unexpectedly, when IAV-GFP was added, the proportion of mCherry-positive cells was significantly increased with a proportion of positive cells between 43 and 84%, depending on the experiment and the time of addition (Figure 3 and Supplementary Figure S1, red hatched bars). The effect was stronger when IAV-GFP was added 1 h prior to IBV-mCherry (3.8-fold increase, Figure 3C) than when added 1 h after (2.1-fold increase, Figure 3B) or simultaneously (2.1-fold increase, Figure 3A). Similar results were obtained in two other experiments (Supplementary Figure S1). Variations in the proportions of positive-cells corresponded to similar variations in fluorescence intensities (Figures 3D–F), suggesting that the replicative capacity is impacted upon co-infection. A concomitant effect on infectivity cannot be excluded.

Therefore, IAV and IBV exert opposite effects on each other’s viral replication upon heterotypic co-infection. While IBV-mCherry infection inhibits IAV-GFP infection, IAV-GFP infection enhances IBV-mCherry infection. To our knowledge, this gain in IBV infection upon IAV co-infection has never been described so far. We therefore focused on this aspect in the following experiments, using the condition which gave the strongest enhancing effect, i.e., infection with IAV 1 h prior to infection with IBV.

### Enhanced IBV Infection Mediated by IAV Is Not Strain Specific

**FIGURE 4:**
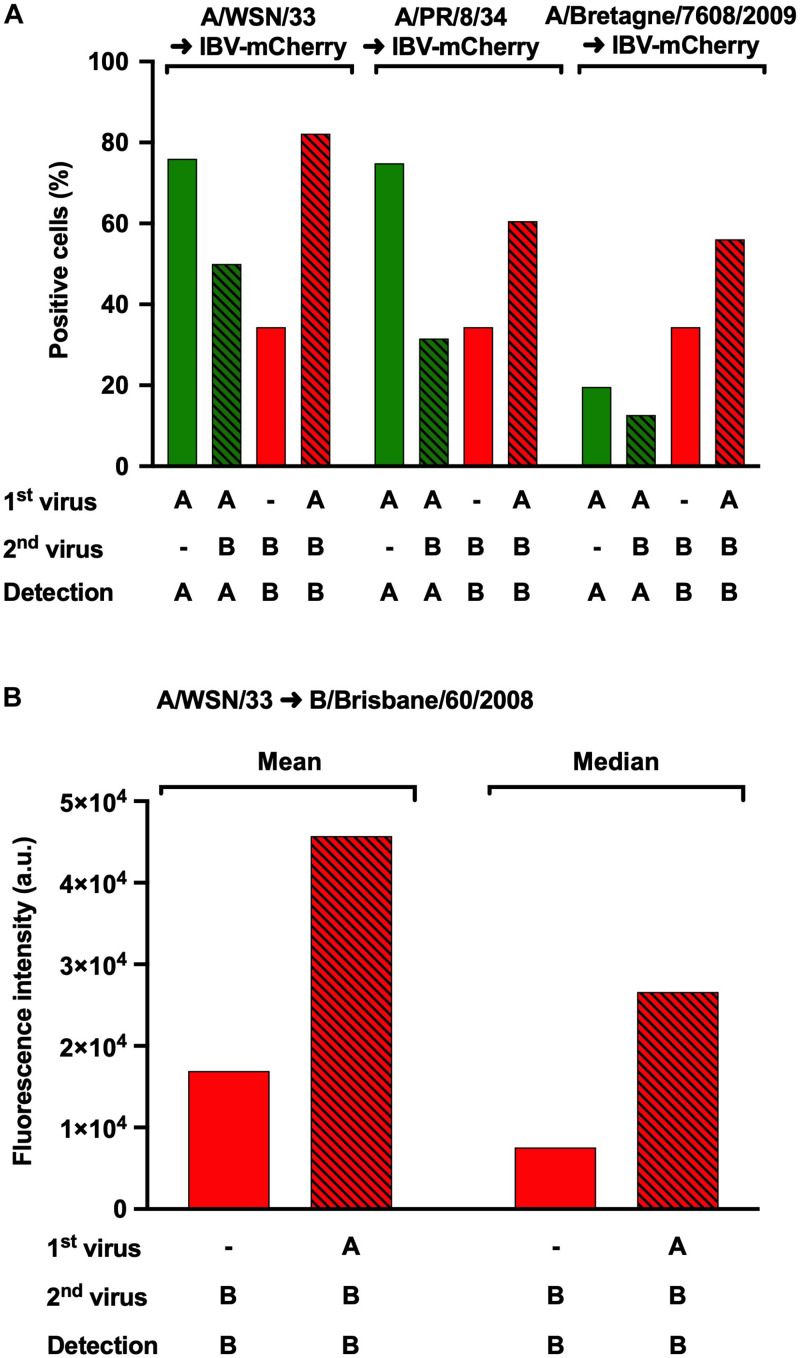
Effect of heterotypic co-infections using different strains of IAV. **(A)** A549 cells were infected with IAV strains A/WSN/33, A/PR/8/34 or A/Bretagne/7608/2009 for 1 h and then infected with IBV-mCherry for 6 h. Infected cells were labeled for the NP protein from IAV and analyzed by flow-cytometry for NP_A_ or mCherry expression. The solid bars represent the proportion of fluorescent cells expressing NP_A_ (in green) or mCherry (in red) upon control infections with one of the indicated IAV strains or IBV-mCherry alone, respectively. The hatched bars represent the proportion of fluorescent cells expressing NP_A_ (in green) or mCherry (in red) upon co-infections. One experiment representative of three independent experiments performed in monoplicate is shown (see Supplementary Figures S2A,B). **(B)** A549 cells were infected for 1 h with A/WSN/33 and then infected with B/Brisbane/60/2008 for 6 h. Infected cells labeled for the NP protein of IBV were detected by flow cytometry. The solid bars represent the mean or median fluorescence intensity upon control infection with B/Brisbane/60/2008 alone. The hatched bars represent the mean or median fluorescence intensity upon co-infection with A/WSN/33 and B/Brisbane/60/2008. One experiment representative of two independent experiments performed in monoplicate is shown (see Supplementary Figure S2C). a.u., arbitrary units.

To determine whether the observed effect was IAV-strain specific, the same protocol as above was used, using A/WSN/33, A/PR/8/34 or A/Bretagne/7608/2009 for the initial IAV infection, followed with a secondary IBV-mCherry infection (Figure 4A). IAV-infected cells were detected following NP_A_ labeling and IBV-infected cells were detected upon mCherry-expression. In control infections, the proportion of NP_A_-positive cells (Figure 4A, green solid bars) was similar for A/WSN/33 (76%) and A/PR/8/34 (75%), but lower for A/Bretagne/7608/2009 (20%). In the presence of IBV-mCherry, the proportion of NP_A_-positive cells was reduced for all three strains (Figure 4A, green hatched bars). In sharp contrast, the proportion of IBV-infected, mCherry-positive cells was increased in the presence of all three IAV strains (Figure 4A, red hatched bars) compared with control infection (Figure 4A, red solid bars). Notably, the increase in mCherry-positive cells was similar in the presence of A/WSN/33, A/PR/8/34, or A/Bretagne/7608/2009 although the proportion of NP_A_-positive cells was lower upon infection with the A/Bretagne/7608/2009 virus, suggesting a less efficient NP_A_-labeling by the monoclonal antibody for the latter. Similar results were obtained in two other experiments (Supplementary Figures S2A,B). These data demonstrate that the increase of IBV infection upon heterotypic co-infection is not IAV-strain specific.

We then asked whether our observations could be reproduced using both a wild-type IAV and a wild-type IBV. A549 cells were infected at a MOI of 3 with A/WSN/33 for 1 h and then with B/Brisbane/60/2008 for 6 h, following the same protocol as in Figure 2C. To detect IBV-infected cells by flow-cytometry, the NP_B_ protein was labeled using a specific monoclonal antibody as described in the “Materials and Methods” section. Because this resulted in a proportion of NP_B_-positive cells close to 100%, we used fluorescence intensity as a read-out to assess the effect of IAV co-infection. The mean and median fluorescence intensities were both increased in co-infected cells (Figure 4B and Supplementary Figure S2C, red hatched bars) compared to cells infected with IBV alone (Figure 4B and Supplementary Figure S2C, red solid bars). This observation provided confirmation that the recombinant reporter viruses represent a reliable tool to study the mutual effects of IAV and IBV upon co-infection.

### Infectious IAV Is Required to Enhance IBV Infection

**FIGURE 5:**
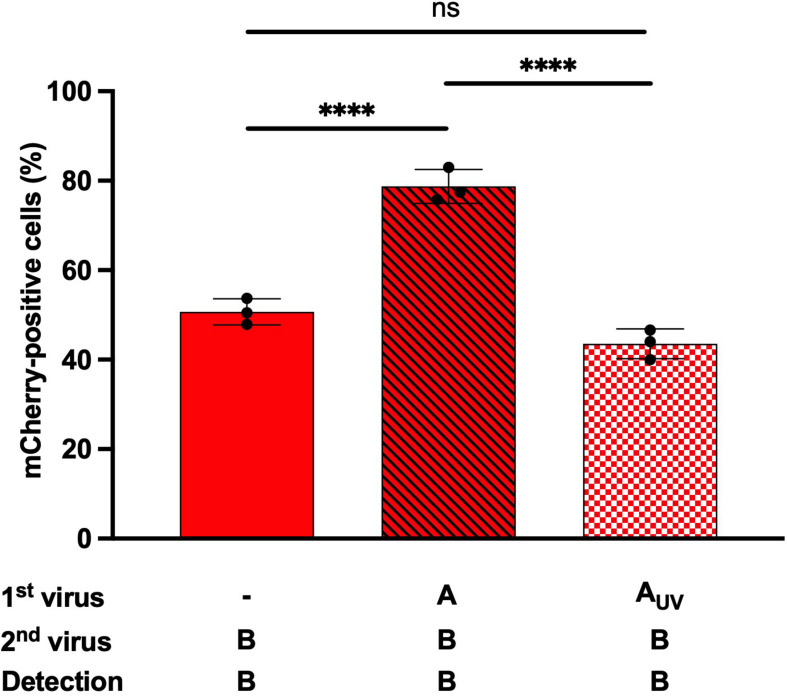
Effect of UV-inactivated IAV on IBV infection. A549 cells were infected for 1 h with IAV-GFP or an UV-inactivated IAV-GFP (IAV-GFP-UV) and then with IBV-mCherry for 6 h and analyzed by flow cytometry. The solid bar represents the proportion of mCherry-positive fluorescent cells upon control infection with IBV-mCherry alone. The hatched bar represents the proportion of mCherry-positive cells upon co-infections. The squared bar represents the proportion of mCherry-positive cells upon co-infection with IAV-GFP-UV and IBV-mCherry. One experiment representative of two independent experiments performed in triplicate is shown (see Supplementary Figure S3). The mean ± S.D. of biological triplicate is shown. Two-way ANOVA test: multiple comparison, Dunn-Sidak test, α = 0.05; ns, not significant; ^****^: adjusted *p* ≤ 0.0001.

We compared an infectious IAV-GFP and a UV-inactivated IAV-GFP in heterotypic co-infections experiments (Figure 5 and Supplementary Figure S3), to assess whether a fully infectious IAV was required to enhance IBV infection, or whether bystander molecule(s) secreted by the MDCK cells during IAV amplification and present in the IAV stock used for co-infection could possibly account for the enhanced IBV infection. The proportion of mCherry-positive cells was increased when cells were co-infected with IBV-mCherry and IAV-GFP (79%, Figure 5, hatched bars) as compared to cells infected with IBV-mCherry alone (51%, Figure 5, solid bars), as expected. In the presence of the UV-inactivated IAV-GFP, no such increase was observed and the proportion of mCherry-positive cells was similar as upon single infection with IBV-mCherry (43%, Figure 5, squared bars). Similar results were obtained in another experiment (Supplementary Figure S3). These results indicate that the enhancement of IBV infection observed upon heterotypic co-infection is not merely due to a soluble factor secreted by infected MDCK cells and present in the IAV stock but requires a replicative IAV.

### IAV Transcription/Replication Steps Are Needed to Enhance IBV Infection

**FIGURE 6:**
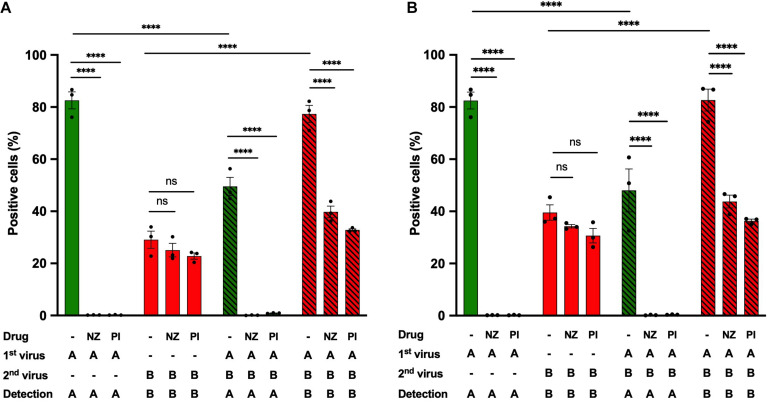
Effect of IAV-specific antiviral compounds on heterotypic co-infections. A549 cells were infected with IAV-GFP for 1 h and then with an IBV-mCherry virus from two different stocks **(A,B)** in the presence or absence of the antiviral compounds nucleozin (1 μM, NZ) or pimodivir (50 nM, PI). At 6 hpi, cells were analyzed by flow cytometry. The solid bars represent the proportion of fluorescent cells expressing GFP (in green) or mCherry (in red) upon control infection with IAV-GFP or IBV-mCherry alone. The hatched bars represent the proportion of fluorescent cells expressing GFP (in green) or mCherry (in red) upon co-infections. One experiment representative of three independent experiments performed in triplicate is shown (see Supplementary Figure S4). The mean ± S.D. of biological triplicates is shown. Three-way ANOVA test: multiple comparison, Tukey test, α = 0.05; ns, not significant; ^****^: adjusted *p* ≤ 0.0001.

To gain further insight into the steps of the IAV life cycle required for IBV infection potentiation, we performed heterotypic co-infection experiments in the presence of antiviral compounds specific to IAV and that do not affect IBV (Figure 6 and Supplementary Figure S4). Nucleozin, by interacting with NP_A_, triggers its aggregation, prevents its nuclear accumulation, and therefore inhibits IAV replication (Kao et al., 2010). Pimodivir targets the PB2 subunit of IAV, prevents its binding to the cap structure, and therefore inhibits cap snatching and IAV transcription (Clark et al., 2014). Both antiviral compounds were used at concentrations corresponding to ∼15-fold the EC_50_ concentration (1 μM for nucleozin and 50 nM for primodivir). They were added separately during the IAV adsorption step and maintained on cells during all the experiment. Two different stocks of IBV-mCherry were used (Figures 6A,B). Upon single infection with IAV-GFP, the proportion of GFP-positive cells in the presence of nucleozin (0.2%) or pimodivir (0.2%) was significantly decreased compared with the control (82%), thus confirming that nucleozin and pimodivir efficiently inhibit IAV replication at these concentrations (Figure 6, green solid bars). Upon single infection with IBV-mCherry, the percentage of mCherry-positive cells (Figure 6, red solid bars) was not significantly different between untreated (29 and 39%, Figures 6A,B) and nucleozin (25 and 34%, Figures 6A,B) or pimodivir (23 and 31%, Figures 6A,B) treated cells, in agreement with the specificity of the two drugs toward IAV.

Upon IAV-IBV co-infection, while for untreated cells the proportion of IBV-mCherry-positive cells was increased (77 and 82%, Figures 6A,B) compared to cells infected with IBV alone (29 and 39%, Figures 6A,B), no such increase was observed in nucleozin- or pimodivir- treated-cells (40–44% and 33–36% mCherry-positive cells in co-infected cells, respectively, compared to 29–39% for cells infected with IBV alone) (Figures 6A,B, compare red hatched and solid bars). Similar results were obtained in two other experiments (Supplementary Figure S4). These results indicate that transcription/replication of IAV or a later step is required to enhance IBV infection upon heterotypic co-infection.

## Discussion

In this study, we developed an original and efficient system to investigate the effects of heterotypic co-infections between IAV and IBV using fluorescent reporter viruses of both viral types. Viral interference is the situation whereby the replication of one virus interferes with the replication of another virus. It has been shown from earlier studies that IBV interferes with IAV replication (Tobita and Ohori, 1979; Mikheeva and Ghendon, 1982; Kaverin et al., 1983; Aoki et al., 1984; Wanitchang et al., 2012). Wanitchang et al. (2012) have shown that infection with a wild-type B/Lee/40 or B/Maryland/2/59 virus inhibited the replication of a recombinant IAV harboring a DsRed reporter gene in the A/PR/8/34 background. However, they did not assess the effect of IAV on IBV infection. Here, we applied a strategy similar to the one used previously to engineer recombinant IAV or IBV from the B/Yamagata lineage (Diot et al., 2016; Biquand et al., 2017) to produce a recombinant mCherry-expressing IBV in a B/Brisbane/60/2008 background (B/Victoria lineage). We set up experimental conditions in which IBV-mCherry infection of A549 cells resulted in the detection of 20–60% of mCherry-positive cells at 6 hpi, which allowed us to assess quantitatively the mutual interference between IAV and IBV.

Using our system to study heterotypic interference, we showed that IAV and IBV exert opposite effects on each other. Indeed, our study confirms previous published data showing that IBV strongly suppressed IAV infection (Tobita and Ohori, 1979; Mikheeva and Ghendon, 1982; Kaverin et al., 1983; Aoki et al., 1984; Wanitchang et al., 2012) but reveals, interestingly, that IAV has the ability to enhance IBV infection. Based on previously published data showing that productive co-infections could only occur if the interval between the two infections did not exceed ∼2 h (Dou et al., 2017), we performed either simultaneous infections or infections 1 h apart. Differences in the kinetics of viral entry can affect the outcome of co-infections. It has been shown from studies in single cells that the entry and fusion of influenza viruses occur rapidly (∼10 min) and that vRNPs are imported into the nucleus in less than 1 h (Dou et al., 2018; Qin et al., 2019). The fact that we observed the same trends whether co-infections were performed simultaneously or whether one virus was added 1 h before or 1 h after the other suggests that a delay in entry kinetics is not a major determinant. The proportion of IAV-GFP-positive cells was decreased 4.8—42-fold upon IBV co-infection, with the strongest effect observed upon simultaneous co-infection. This is in agreement with previous data showing that if IAV infection occurred 2 h prior to infection with IBV, IBV could no longer suppress IAV replication (Wanitchang et al., 2012). The mechanism allowing the inhibition of IAV infection upon IBV co-infection was shown to involve the binding of NP_B_ to NP_A_ and the subsequent inhibition of IAV genome transcription/replication (Wanitchang et al., 2012; Jaru-ampornpan et al., 2014). While it has been shown that NP_B_ expression suppressed IAV polymerase activity, addition of NP_A_ did not significantly inhibit IBV polymerase activity (Wanitchang et al., 2012; Baker et al., 2014). This is consistent with our observation of an absence of a symmetrical negative interference of IAV on IBV infection. On the contrary, in our experiments the proportion of IBV-mCherry-positive cells was enhanced 1.8—2.5-fold upon IAV co-infection. The gain in IBV infection upon co-infection with IAV was observed with different stocks of IBV-mCherry, with wild-type IBV, and with various strains of IAV from the H1N1 subtype, therefore excluding that the observed effect is IBV-stock-dependent or IAV-strain-dependent.

We then tried to explore by which mechanisms IAV increases IBV infection. Enhancement of IBV-mCherry infection was the strongest when IAV infection was initiated 1 h before IBV-mCherry infection, however, it was also observed when IAV was added simultaneously and even 1 h after IBV-mCherry infection. Taken together with the fact that IAV infection increased not only the percentage of mCherry-positive cells, but also the mean and median mCherry fluorescence intensities which reflect the level of expression of the PB2 viral protein, a likely hypothesis is that IAV exerts its enhancing effect at the level of IBV transcription/replication. By using a UV-inactivated IAV-GFP or antiviral compounds targeting specific steps of the IAV life cycle, we showed that enhancement of IBV infection was no longer observed when IAV transcription/replication was inhibited. UV treatment induces pyrimidine dimers formation in the viral RNA genome and results in a loss of infectivity of UV-treated viruses (Delrue et al., 2012). UV-inactivated viruses can still to some extent interact with their target cells, e.g., be endocytosed, stimulate PPR, induce receptor signaling (Delrue et al., 2012). However, these properties do not account for the observed effect of IAV on IBV infection, as the enhancement of IBV infection was no longer observed with UV-inactivated IAV. This suggests that (i) the process of IAV transcription/replication *per se* and/or (ii) a viral protein resulting from the transcription/replication of the IAV genome, and/or (iii) a late stage of the IAV cycle may be involved in the positive interference on IBV. Our experimental design and read-out (GFP expression as a proxy for PB2 expression at 6 phi) is in favor of (i) and/or (ii), although (iii) cannot be completely excluded.

Previous publications have shown that the IAV polymerase complex can recognize the promoter of IBV suggesting a functional compatibility between the type A polymerase machinery and the type B promoter (Crescenzo-Chaigne et al., 1999). Moreover, despite a limited compatibility among the polymerase subunits and nucleoprotein of both types (Iwatsuki-Horimoto et al., 2008), it was shown that none of the IAV proteins was able to block IBV polymerase activity (Wanitchang et al., 2012). It is therefore tempting to speculate that, upon co-infection, one or several IAV proteins could enhance transcription/replication of IBV vRNAs. The IAV proteins involved could correspond to the polymerase complex itself and/or other proteins imported back in the nucleus during IAV transcription/replication, such as NS1 (non-structural protein 1). Indeed, NS1 has been described to positively regulate transcription/replication of the IAV genome and translation of viral mRNAs (for a review on NS1, see Hale et al., 2008). NS1 proteins of IAV and IBV share limited sequence homology, yet both have the ability to counteract the type 1 and type 3 IFN responses and are essential for efficient viral growth (Dauber et al., 2004, 2006; Hai et al., 2008; Patzina et al., 2017; Nogales et al., 2019). Despite homologous functions, NS1_B_ has been shown to not fully complement the functions of NS1_A_ in the context of recombinant IAV viruses expressing heterotypic NS1, suggesting that other functions specific for NS1_A_ are not present in NS1_B_ (Nogales et al., 2019). One hypothesis is that, in the context of IAV-IBV co-infections, IAV-NS1 could exert effects similar to IAV RNAs on IBV RNAs and increase transcription/replication or translation of the IBV genome. This could be mediated either through an increase of the activity of the IAV polymerase or of the IBV polymerase on the IBV genome. Another mechanism could involve the complementation of IBV-defective particles by IAV. As suggested by the physical to infectious particles ratio, a higher proportion of defective particles is present in the IBV-mCherry than in the IAV-GFP viral stock. It has been shown for IAV that the susceptibility to superinfection was determined by the level of semi-infectious particles in the population (Sun and Brooke, 2018). In a similar way, semi-infectious particles in the IBV population could be rescued by the expression of IAV proteins.

Using fluorescent-reporter viruses, we confirmed previous published data regarding inhibition of IAV infection by IBV. We also brought new data on influenza heterotypic co-infections showing that IBV infection can be enhanced upon IAV co-infection, and more strongly so if IAV is added prior to IBV. We also showed that IAV transcription/replication steps were required to enhance IBV infection. We therefore suggest that the IAV polymerase complex, while being inhibited by NP_B_ on type A vRNP, could use type B vRNA as a template for transcription/replication and/or that another IAV protein could enhance the activity of IBV or IAV polymerase complex on the IBV promoter. Co-infection experiments using various IBV strains (including the B/Yamagata lineage) and IAV subtypes (including the circulating H3N2 subtype) could help further elucidate the underlying mechanism in the future.

Extrapolation from our experimental evidence for enhancement of IBV infection upon co-infection with IAV to its significance in a natural setting should be cautious. In natural infections, the MOI increases as infection spreads and locally at the peak of infection it may reach high values that allow co-infections (Tao et al., 2014, 2015). It is tempting to speculate that influenza heterotypic co-infections could alter disease severity and possibly make some IBV infections become more severe. Our system could further be adapted to study co-infections between influenza viruses and other respiratory viruses, in humans or in other species such as swine. In the context of the COVID-19 pandemic, it is of major importance to study the impact of influenza and SARS-CoV-2 co-infections on disease severity and on the epidemiology of both viruses.

## Data Availability Statement

The raw data supporting the conclusions of this article will be made available by the authors, without undue reservation, to any qualified researcher.

## Author Contributions

NM, SM, and NN conceived and designed the experiments and wrote the original draft of the manuscript. NM and SM performed the experiments and analyzed the data. SW provided expertise and feedback and secured funding. All authors contributed to the article and approved the submitted version.

## Conflict of Interest

The authors declare that the research was conducted in the absence of any commercial or financial relationships that could be construed as a potential conflict of interest.

## Supplementary Material

Supplementary Figure 1 Effect of heterotypic co-infections between IAV and IBV. A549 cells were co-infected, either simultaneously **(A,B)** or sequentially **(C–F)**, with IAV-GFP and IBV-mCherry at a MOI of 3 PFU/cell for each virus and analyzed 6 h after the addition of the last virus using flow cytometry. The solid bars represent the proportion of fluorescent cells expressing GFP (in green) or mCherry (in red) upon control infection with IAV-GFP or IBV-mCherry alone. The hatched bars represent the proportion of fluorescent cells expressing GFP (in green) or mCherry (in red) upon co-infections. **(A,B)** Simultaneous co-infection with IAV-GFP and IBV-mCherry viruses. **(C,D)** Primary infection with IBV-mCherry followed 1 hpi with IAV-GFP infection. **(E,F)** Primary infection with IAV-GFP followed 1 hpi with IBV-mCherry infection. The mean ± S.D. of biological triplicates is shown. Two-way ANOVA test: multiple comparison, Dunn-Sidak test, α = 0.05; ^∗∗∗^: adjusted *p* = 0.0001; ^****^: adjusted *p* ≤ 0.0001.

Supplementary Figure 2 Effect of heterotypic co-infections using different strains of IAV. **(A,B)** A549 cells were infected with IAV strains A/WSN/33, A/PR/8/34 or A/Bretagne/7608/2009 for 1 h and then infected with IBV-mCherry for 6 h. Infected cells were labeled for the NP protein from IAV and analyzed by flow-cytometry for NP_A_ or mCherry expression. The solid bars represent the proportion of fluorescent cells expressing NP_A_ (in green) or mCherry (in red) upon control infections with one of the indicated IAV strains or IBV-mCherry alone, respectively. The hatched bars represent the proportion of fluorescent cells expressing NP_A_ (in green) or mCherry (in red) upon co-infections. **(C)** A549 cells were infected for 1 h with A/WSN/33 and then infected with B/Brisbane/60/2008 for 6 h. Infected cells labeled for the NP protein of IBV were detected by flow cytometry. The solid bars represent the mean or median fluorescence intensity upon control infection with B/Brisbane/60/2008 alone. The hatched bars represent the mean or median fluorescence intensity upon co-infection with A/WSN/33 and B/Brisbane/60/2008. a.u., arbitrary units.

Supplementary Figure 3 Effect of UV-inactivated IAV on IBV infection. A549 cells were infected for 1 h with IAV-GFP or an UV-inactivated IAV-GFP (IAV-GFP-UV) and then with IBV-mCherry for 6 h and analyzed by flow cytometry. The solid bar represents the proportion of mCherry-positive fluorescent cells upon control infection with IBV-mCherry alone. The hatched bar represents the proportion of mCherry-positive cells upon co-infections. The squared bar represents the proportion of mCherry-positive cells upon co-infection with IAV-GFP-UV and IBV-mCherry. The mean ± S.D. of biological triplicate is shown. Two-way ANOVA test: multiple comparison, Dunn-Sidak test, α = 0.05; ns, not significant; ^****^: adjusted *p* ≤ 0.0001.

Supplementary Figure 4 Effect of IAV-specific antiviral compounds on heterotypic co-infections. A549 cells were infected with IAV-GFP for 1 h and then with an IBV-mCherry virus from two different stocks [**(A,C)** for the stock used in Figure 6A and **(B,D)** for the stock used in Figure 6B] in the presence or absence of the antiviral compounds nucleozin (1 μM, NZ) or pimodivir (50 nM, PI). At 6 hpi, cells were analyzed by flow cytometry. The solid bars represent the proportion of fluorescent cells expressing GFP (in green) or mCherry (in red) upon control infection with IAV-GFP or IBV-mCherry alone. The hatched bars represent the proportion of fluorescent cells expressing GFP (in green) or mCherry (in red) upon co-infections. The mean ± S.D. of biological triplicates is shown. Three-way ANOVA test: multiple comparison, Tukey test, α = 0.05; ns: not significant; ^****^: adjusted *p* ≤ 0.0001.
