# Supplementary material for: Influenza B Virus Infection Is Enhanced Upon Heterotypic Co-infection With Influenza A Virus
Source: Front Microbiol. 2021 Feb 25;12:631346. doi: 10.3389/fmicb.2021.631346 (PMC7947630; doi:10.3389/fmicb.2021.631346)
Supplement: Supplementary Figure 1 — Effect of heterotypic co-infections between IAV and IBV. A549 cells were co-infected, either simultaneously (A,B) or sequentially (C–F), with IAV-GFP and IBV-mCherry at a MOI of 3 PFU/cell for each virus and analyzed 6 h after the addition of the last virus using flow cytometry. The solid bars represent the proportion of fluorescent cells expressing GFP (in green) or mCherry (in red) upon control infection with IAV-GFP or IBV-mCherry alone. The hatched bars represent the proportion of fluorescent cells expressing GFP (in green) or mCherry (in red) upon co-infections. (A,B) Simultaneous co-infection with IAV-GFP and IBV-mCherry viruses. (C,D) Primary infection with IBV-mCherry followed 1 hpi with IAV-GFP infection. (E,F) Primary infection with IAV-GFP followed 1 hpi with IBV-mCherry infection. The mean ± S.D. of biological triplicates is shown. Two-way ANOVA test: multiple comparison, Dunn-Sidak test, α = 0.05; ∗∗∗: adjusted p = 0.0001; ****: adjusted p ≤ 0.0001. [file Data_Sheet_1.PDF]

Figure S1

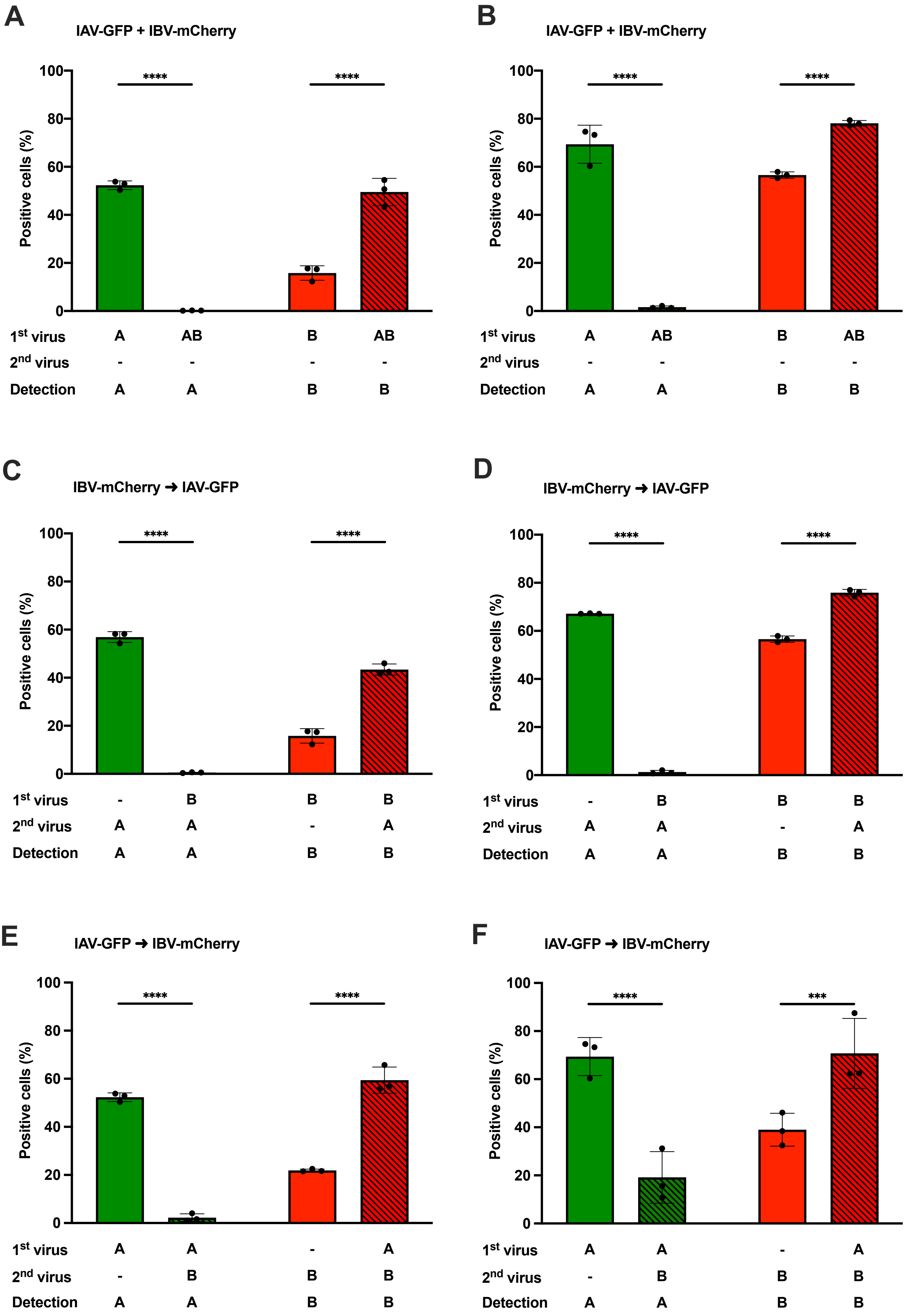
A
IAV-GFP + IBV-mCherry
Positive cells (%)
****
****
1st virus A AB B AB
2nd virus - - - -
Detection A A B B
B
IAV-GFP + IBV-mCherry
Positive cells (%)
****
****
1st virus A AB B AB
2nd virus - - - -
Detection A A B B
C
IBV-mCherry ➔ IAV-GFP
Positive cells (%)
****
****
1st virus - B B B
2nd virus A A - A
Detection A A B B
D
IBV-mCherry ➔ IAV-GFP
Positive cells (%)
****
****
1st virus - B B B
2nd virus A A - A
Detection A A B B
E
IAV-GFP ➔ IBV-mCherry
Positive cells (%)
****
****
1st virus A A - A
2nd virus - B B B
Detection A A B B
F
IAV-GFP ➔ IBV-mCherry
Positive cells (%)
****
***
1st virus A A - A
2nd virus - B B B
Detection A A B B

# Figure S2

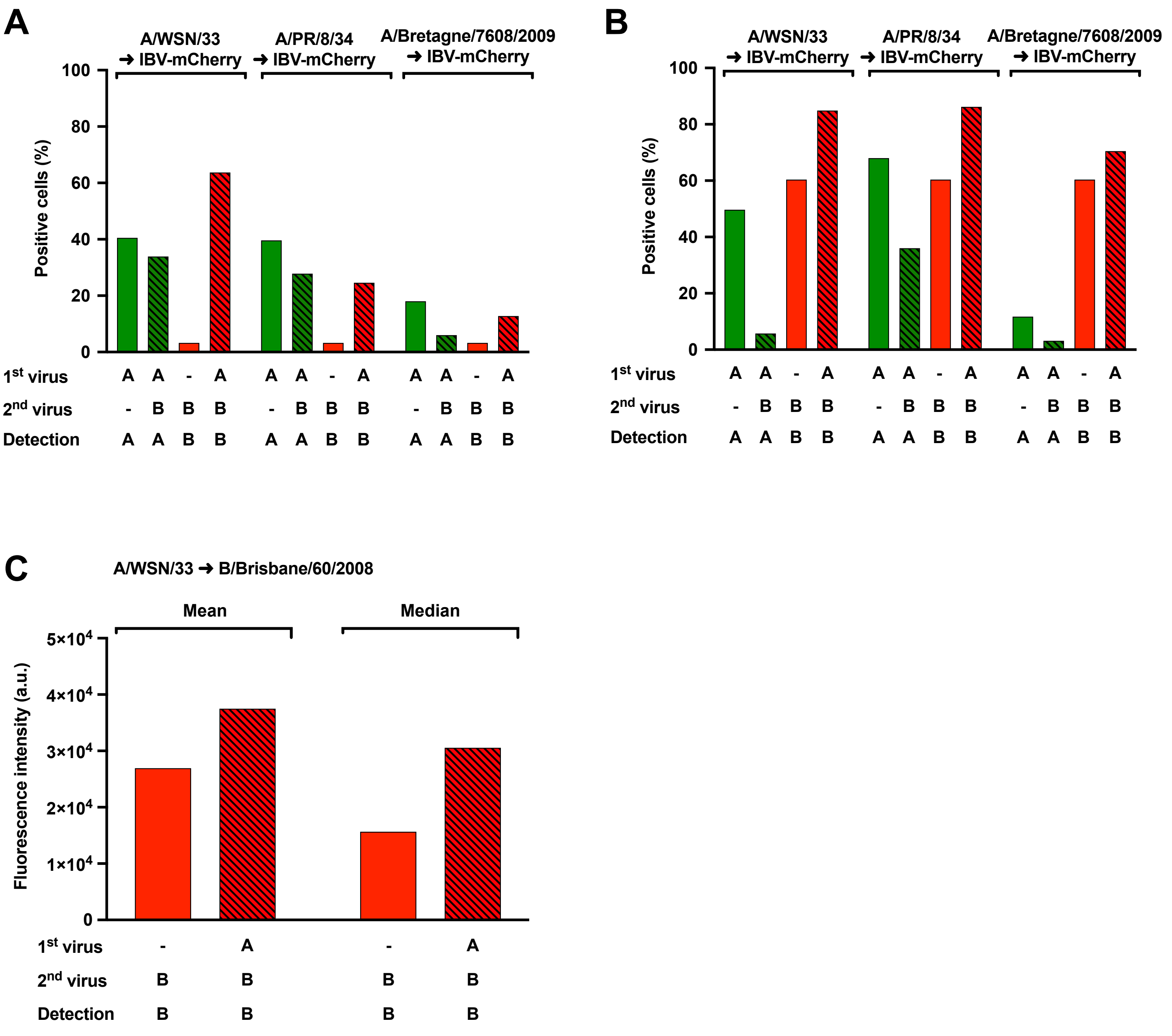
A
A/WSN/33 ➔ IBV-mCherry
A/PR/8/34 ➔ IBV-mCherry
A/Bretagne/7608/2009 ➔ IBV-mCherry
Positive cells (%)
0
20
40
60
80
100
1st virus
2nd virus
Detection
B
A/WSN/33 ➔ IBV-mCherry
A/PR/8/34 ➔ IBV-mCherry
A/Bretagne/7608/2009 ➔ IBV-mCherry
Positive cells (%)
C
A/WSN/33 ➔ B/Brisbane/60/2008
Mean
Median
Fluorescence intensity (a.u.)
0
1×10^4
2×10^4
3×10^4
4×10^4
5×10^4
1st virus
2nd virus
Detection

# Figure S3

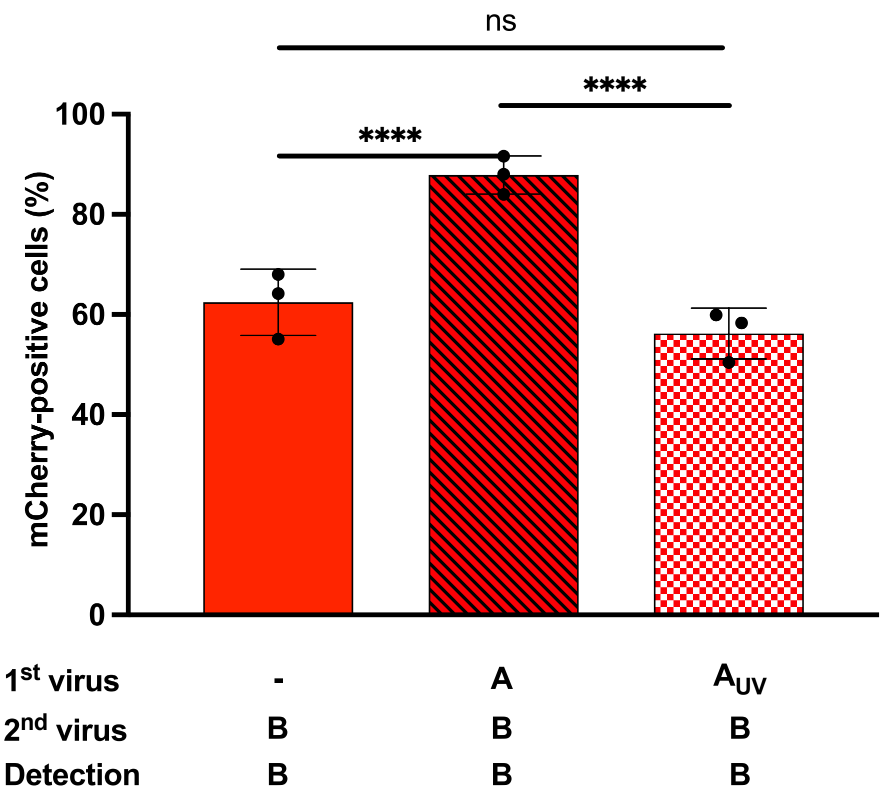

ns
****
****
100
80
60
40
20
0
mCherry-positive cells (%)
1st virus
-
A
AUV
2nd virus
B
B
B
Detection
B
B
B

Figure S4

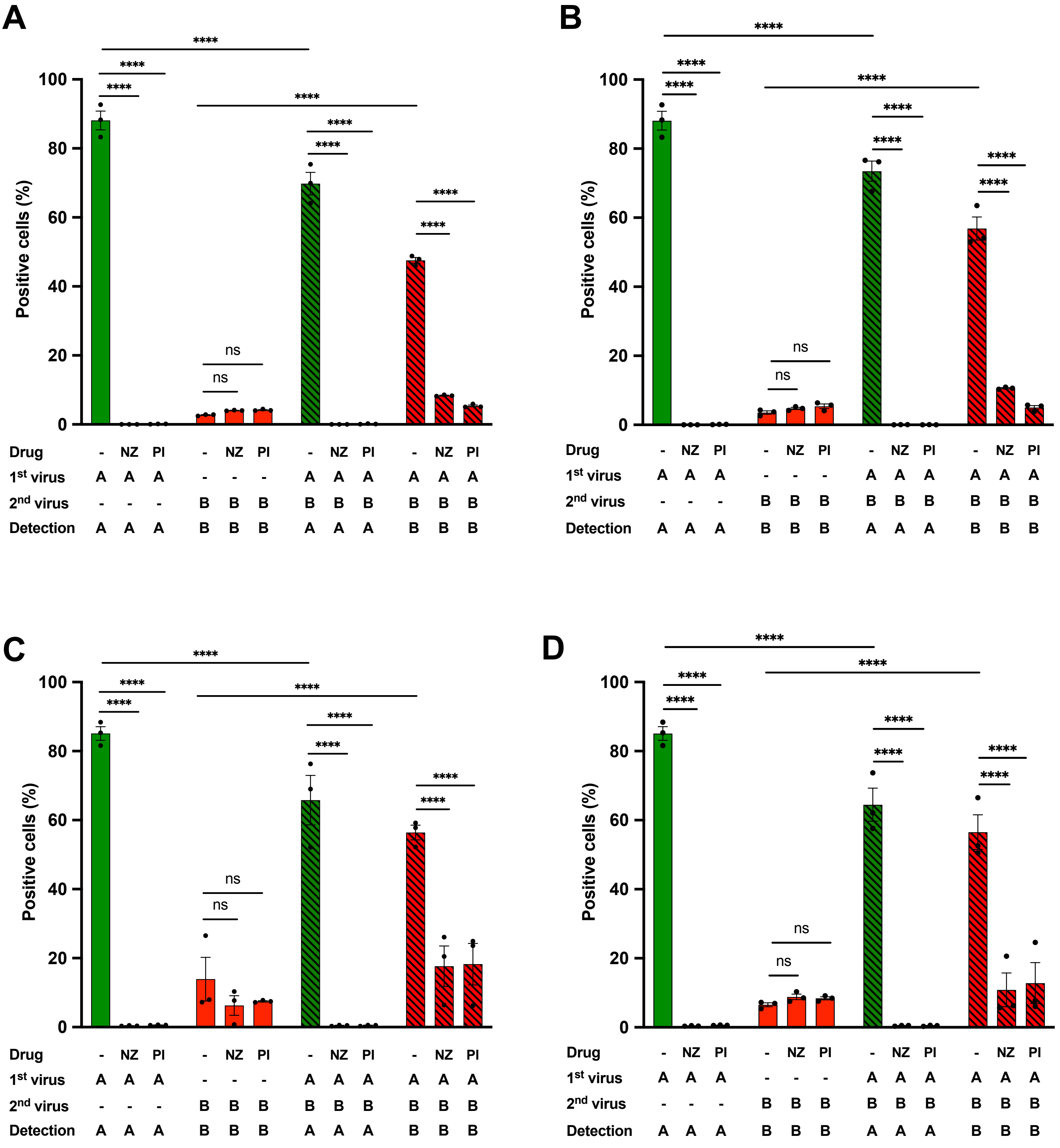
A
B
C
D
Positive cells (%)
0
20
40
60
80
100
****
ns
Drug - NZ PI - NZ PI - NZ PI - NZ PI
1st virus A A A - - - A A A A A A
2nd virus - - - B B B B B B B B B
Detection A A A B B B A A A B B B
